# Rapid spread of double East- and West-African *kdr* mutations in wild *Anopheles coluzzi* from Côte d’Ivoire

**DOI:** 10.12688/wellcomeopenres.15105.1

**Published:** 2019-02-15

**Authors:** Chouaïbou Seïdou Mouhamadou, Prisca Bédjou N’Dri, Behi Kouadio Fodjo, Christabelle Gba Sadia, France-Paraudie Kouadio Affoue, Benjamin Guibehi Koudou

**Affiliations:** 1 Centre Suisse de Recherches Scientifiques, Abidjan, Cote d'Ivoire; 2 Department of Entomology and Plant Pathology, North Carolina State University, Raleigh, NC, 27695-7508, USA; 3 Department of Epidemiology and Public Health, Swiss Tropical and Public Health Institute, University of Basel Switzerland, Basel, Switzerland

**Keywords:** Vector control, Insecticides, Long lasting insecticidal bednet, Indoor residual spraying, insecticide resistance, knockdown resistance

## Abstract

Malaria morbidity and mortality rates in Sub-Saharan Africa are increasing. The scale-up of long-lasting insecticidal nets and indoor residual spraying have been the major contributors to the decrease of malaria burden. These tools are now threatened by insecticide resistance in malaria vectors, which is spreading dramatically. After two different real-time polymerase chain reaction molecular characterizations carried out on 70 mosquitoes sampled in the locality of Elibou in southern Côte d’Ivoire, results revealed that 9 mosquitoes from *Anopheles coluzzi* harbored the double East- and West-African knockdown resistance mutations. In the previous year, only 1 mosquito out of 150 sampled from 10 regions of the country had the same genotype. These results show the rapid spread of insecticide resistance in malaria vectors and highlight the urgent need to diversify the methods of vector control in order to avoid the failure of insecticide-based vector control tools which may favor malaria fatalities.

## Background

Malaria morbidity and mortality rates in Sub-Saharan Africa are increasing, with the number of World Health Organization (WHO)-estimated cases reaching 219 million, with 435 000 associated deaths, in 2017 ^1^ comparing to the 216 million scored in 2016 which had already increased for about 5 million cases over 2015 ^2^. Malaria prevention relies on vector control using insecticides, either by indoor residual spraying (IRS) or in long-lasting insecticide-impregnated mosquito bed nets (LLINs). The efficacy of these measures depends primarily on the susceptibility of the malaria vectors to insecticides. An estimated 663 million cases of malaria have been averted in sub-Saharan Africa between 2000 and 2015 as a result of the scale-up of malaria control interventions, of which 68%, 22% and 10% were attributed to LLINs, artemisinin-based combination therapy (ACT) and IRS, respectively. Unfortunately, despite these efforts, the number of resistant mosquito populations is increasing dramatically, and the efficacy of pyrethroids (the most commonly used insecticide class) is decreasing ^3, 4^, which translates to an increase in malaria cases ^1, 2^.

The two major causes of resistance to pyrethroids include alterations in the target site (knockdown resistance ( *kdr*)) and increases in the rate of insecticide metabolism by enzymes in various P450 families ^5^. This *kdr* occurs due to mutations in the para-gated sodium channel gene. In *Anopheles gambiae*, two *kdr* mutations (1014F ^6^ and 1014S ^7^) have been identiﬁed at the same codon.

Studies aimed at estimating the frequency of these mutations across Africa have shown that the 1014F *kdr* mutation has spread from West Africa ^8– 11^ to East Africa ^12– 14^, and the 1014S *kdr* mutation has spread from East Africa to Central and West Africa ^15, 16^. In Côte d’Ivoire, resistance to insecticides used for vector control is prevalent ^17, 18^, involving multiple mechanisms ^19^. So far, only two cases of East African *kdr* (1014S) have been reported, both a few years ago, in Côte d’Ivoire; the first by Chouaibou *et al.*, ^20^ and the second by Fodjo *et al.*, ^18^, each on individual *An. gambiae* mosquitoes.

Further follow-up studies carried out recently helped us to describe the recent and rapid spread of 1014S mutation in Côte d’Ivoire. Interestingly, our key findings demonstrated that development of the 1014S mutation occurs exclusively in mosquitoes that already have the 1014F mutation. *An. gambiae* mosquitoes bearing both *kdr* mutations are described in detail below.

## Methods

### Mosquitoes

The mosquitoes used in this study were collected as part of the large bionomic study of malaria transmission in the locality of Elibou (5°40′57″N; 4°30′30″W) in South Côte d’Ivoire. Sampling was done in the larval stage in several breeding sites. Larvae were evenly pooled together and reared to the adult stage at the *Centre Suisse de Recherches Scientifiques* (CSRS) insectarium under standard conditions (temperature of 25–27°C and 70–90% relative humidity).

### Genotyping of mosquitoes

Genomic DNA was extracted from 70 adult mosquitoes using the MegaZorb® DNA Mini-Prep Kit according to the manufacturer instructions (Promega Corporation, USA). The identification of *An. gambiae* complex members was made by short interspersed element (SINE)-PCR according to the methods described by Santolamazza *et al.* ^21^. The East- and West-African knockdown resistance genes were characterized using a triplex assay, optimized by Mavridis *et al.* ^22^ from Bass *et al.* ^23^ for simultaneously detecting the wildtype L1014 and the *kdr* mutations 1014F and 1014S in the same reaction. The reaction was performed on a Bio-Rad CFX96 Real-Time System using DNA extractions of individual mosquitoes in 10-µl reaction volumes. Each probe was labelled with a different ﬂuorescent dye: HEX for wildtype L1014 (CTTACGACTAAATTTC), FAM for 1014F (ACGACAAAATTTC), and Atto for 1014S (ACGACTGAATTTC). The cycling conditions used were 50°C for 15 min, 95°C for 3 min, and 40 cycles of 95°C for 3 s and 60°C for 30 s, allowing a PCR run of approximately 70 min. Genotypes appeared during amplification as three-color curves.

At the end of this initial molecular analysis, nine mosquitoes presented the 1014S mutation. Surprisingly, these same mosquitoes also presented the 1014F mutation. To confirm genotyping results, the DNA extractions of the same mosquitoes were further used in TaqMan assays ^23^ to characterize 1014F and 1014S mutations. In each of the East- *kdr* and West- *kdr* assay, two probes labelled with fluorochromes FAM (ID for Lifetech: AHGI2PM) and HEX (ID for Lifetech: AHAA5AD) were used to detect the mutant alleles and the wild type susceptible allele, respectively. The reaction was performed on the Bio-Rad CFX96 Real-Time qPCR thermal cycler in 10-μl reactions volumes, including master mix, primer/probe, and water. The thermal cycle parameters were 10 min at 95°C, then, 40 cycles of 10 s at 95°C and 45 s at 60°C. Genotypes were determined after real-time amplification from dual-color scatter plots using Bio-Rad CFX Manager 3.1 software.

## Results

**Figure 1.**
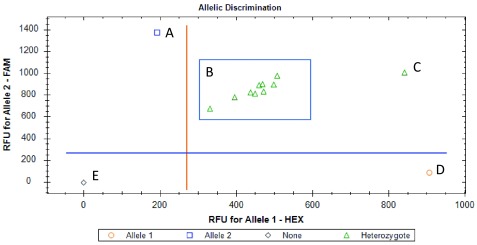
East African *kdr* genotype of wild Elibou *Anopheles coluzzi* population. The ‘A’, ‘C’, ‘D’ and ‘E’ on the figure are the positive controls respectively for the East- *kdr* homozygous mutant allele, heterozygous mutant/susceptible allele, homozygous susceptible allele and blank. Nine mosquitoes (‘B’) displayed the heterozygous

**Figure 2.**
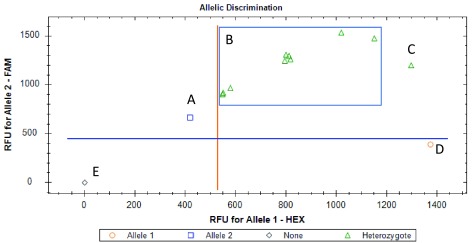
West African *kdr* genotype of wild Elibou *Anopheles coluzzi* population. The ‘A’, ‘C’, ‘D’ and ‘E’ on the figure are the positive controls respectively for the East- *kdr* homozygous mutant allele, heterozygous mutant/susceptible allele, homozygous susceptible allele and blank. The same nine mosquitoes of Figure 1 displayed the heterozygous (‘B’) genotype. We have not quantified the DNA in extracted samples. A low quantity of DNA in the West- *kdr* homozygous positive control might explain the low signal observed for A.

The results obtained with the TaqMan assays and the triplex PCR assays were in agreement. Nine out of 70 *An. gambiae* mosquitoes (12.85%) were found to harbor concurrently both the 1014S *kdr* mutation and the 1014F *kdr* mutation ( Figure 1 and Figure 2). All mosquitoes used in analyses were identified as *An. gambiae coluzzi*. None of the mosquitoes were found to have only the 1014S *kdr* mutation. Raw data are provided on OSF ^24^.

## Discussion and conclusion

In the current study, 9 out of 70 the species of *Anopheles coluzzi* collected in one locality of Elibou in Côte d’Ivoire appeared to have the double East- and West-African *kdr* mutations, whereas just 1 year ago, only 1 mosquito out of 150 sampled across country had the same genotype. These results could be found too preliminary as only one location has been sampled and the change is only observed between two time points. Nevertheless, this should be taken seriously since as the phenotypic consequences and thus importance of the combination of the mutations in the populations is unknown. We have not provided data for the *kdr* 1014F as this is part of another study. Overall, the present study confirms the general trend of intensification and propagation of resistance phenomena. The risks and consequences for vector control and malaria burden are widely documented. WHO ^25^ reported in 2012 that coverage with LLINs and IRS in the WHO African Region was estimated to avert approximately 220,000 deaths among children under 5 years annually. If pyrethroids were to lose most of their efficacy, more than 55% of the benefits of vector control would be lost, leading to approximately 120 000 deaths that could not be averted. To remedy to this, it was found necessary by some companies to reformulate some active ingredients previously used exclusively in agriculture. This is the case for the neonicotinoids, reformulated by Bayer under the name of Fludora, composed of clothianidin and deltamethrin, or by Sumitomo under the name of Sumishield, composed of clothianidin. Neonicotinoids exhibit a mode of action that is completely different to the one appearing when public health insecticides are used. Neonicotinoids act by selectively targeting the invertebrate nicotinic acetylcholine receptor (nAChR) and disrupting excitatory cholinergic neurotransmission leading to paralysis and death ^26^. Given the massive use of neonicotinoids in agriculture ^18, 27^, their medium-to-long-term efficacy on vector populations remains questionable. An upcoming study have shown that wild populations of *An. gambiae* from agricultural areas of Cote d'Ivoire are already resistant to neonicotinoids (Chouaibou *et al.*, Submitted). This dilemma reinforces the idea that the reformulation of agricultural insecticides for public health application is not necessarily the right solution for vector control, although it may appear as a transitional solution. Other solutions include the development of new insecticidal molecules dedicated exclusively to vector control in the public health sector, as described in the goal of the Innovative Vectors Control Consortium for the next 3 to 5 years. Other non-chemical methods of vector control should also be considered for the future. Given the huge emphasis on chemical-based control tools, people may think that chemical-based control strategies are the only way to overcome malaria vectors, while we are confident that the best approach is the integrated vector management strategy that includes all effective and available methods. The current study highlights the rapid spread of insecticide resistance in malaria vectors, which can lead to insecticide-based vector control failure and huge fatalities on malaria burden. Thus, collective awareness is essential. Vector control interventions must be rethought and reviewed overall; reflections must be made at all stages, and chemical control must not be seen as the ultimate solution.

## Data availability

Output data from the genotyping of mosquitoes is available on OSF. DOI: https://doi.org/10.17605/OSF.IO/9BDT7 ^24^.

Data are available under the terms of the Creative Commons Zero "No rights reserved" data waiver (CC0 1.0 Public domain dedication).
