# Supplementary material for: Rapid spread of double East- and West-African kdr mutations in wild Anopheles coluzzi from Côte d’Ivoire
Source: Wellcome Open Res. 2019 Feb 15;4:31. [Version 1] doi: 10.12688/wellcomeopenres.15105.1 (PMC6468708; doi:10.12688/wellcomeopenres.15105.1)
Supplement: Peer review history

# Peer review: Reviewer response for version 1

**DOI:** 10.21956/wellcomeopenres.16481.r35376

**Published:** 2019-05-10

**Authors:** Penelope A. Hancock

**Affiliations:** 1 Li Ka Shing Centre for Health Information and Discovery, University of Oxford, Oxford, UK

The paper is clearly written and the data, analytical methods and results are well explained. I agree that the observation of the double mutation in individual mosquitoes is interesting and warrants further study. However, I think the interpretation drawn from the results presented needs to be re-thought, because the paper does not present sufficient evidence that the frequency of the double East and West African kdr mutation is increasing. Referring to the statement in the first section of the Discussion and conclusion “In the current study, 9 out of 70 of the species of *Anopheles coluzzi *collected in one locality of Elibou in Cote d’Ivoire appeared to have the double East and West African kdr mutations, whereas just 1 year ago, only 1 mosquito out of 150 sampled across the country had the same genotype”. Can the authors be more specific about where the 150 mosquitoes from the previous year were collected and what species they were? A robust statement about whether resistance is spreading would really require more samples, both longitudinally and from multiple locations.

 At present, the results need to be stated in a more qualitative sense, explicating the reasons why a proportion of 9 out of 70 can be considered as alarming, given prior observations.

 I should add that I am not sufficiently qualified to asses the soundness of the molecular analyses presented.

I have read this submission. I believe that I have an appropriate level of expertise to confirm that it is of an acceptable scientific standard, however I have significant reservations, as outlined above.

# Peer review: Reviewer response for version 1

**DOI:** 10.21956/wellcomeopenres.16481.r35116

**Published:** 2019-04-16

**Authors:** Lizette L. Koekemoer

**Affiliations:** 1 Wits Research Institute for Malaria, Faculty of Health Sciences, School of Pathology, University of the Witwatersrand, Johannesburg, South Africa

This study reports on the spread of the kdr-resistant mutations in Cote d’Ivoire.

 The work is clearly written and the following are more specific comments to the authors.

 Title is misleading as this is mainly a pilot study with a small sample size and although the frequency of the mutation seems to have increased, the “rapid spread” would need additional sampling from multiple sites in the country. Title should be revised.

 Spelling of *An. coluzzii*, should be corrected in the manuscript and there is no need to call the species *An. gambiae coluzzii*.

 Background section: Authors' can also rephrase to indicate the personal protection the nets will provide in addition to the killing effect. The increase in malaria cases with an increase in pyrethroid resistance can be mitigated with the use of insecticide resistance management plan. This can be added to explain the sentence stated that it will translate to an increase in malaria cases.

 The authors should use the formal naming of the mutations: **L**1014F and **L**1014S.

 Methods section: indicate how many breeding sites are “several”? Difficult to interpret if the sample size of 70 is from 2 or 50 breeding sites.

 General comments: Fludora and Sumishield are trade names and should be indicated with a “®“

 Are there indications that the population of An. coluzzii with two mutations have an increase in resistance intensity on pyrethroids? What was the resistance on pyrethroids from earlier years, has this changed?

I have read this submission. I believe that I have an appropriate level of expertise to confirm that it is of an acceptable scientific standard.

# Peer review: Reviewer response for version 1

**DOI:** 10.21956/wellcomeopenres.16481.r34886

**Published:** 2019-03-12

**Authors:** Stephen Magesa

**Affiliations:** 1 VectorLink Mozambique Project, Abt Associates Inc. Maputo, Maputo, Mozambique

Title

 The title sounds appropriate, though the initial word “Rapid” in the title referring to the pace at which the double mutations are spreading has been measured and found to be high depicts a misrepresentation. A better word could be found to replace this one. Or else omitting it altogether does no harm to the title. 

 The work is very clearly and accurately presented using simple language. It also cites most of the current literature on the particular subject. Very pertinent being recent work in the same country and other neighbouring countries in the region. However, I have picked a few issues around the way the results are presented interpreted and need for further verification of the results. I would like therefore to raise the following concerns and some additional comments that the authors may wish to use for improving the article:

 The work is technically sound since the laboratory techniques employed in the analysis includes the standard methodologies that are current in the subject. However, the design could have been better by sampling from more areas and if possible in all the ten regions where the initial study by Chouaïbou et al. (2017) ^1^ were sampled. This study is based on samples from only one locality which we may not know whether is a stronghold for these combined gene mutations or not. This is stated with the understanding that the current study is a follow-on of earlier studies by Chouaïbou et al. (2017) ^1^ and Fodjo et al. (2018) ^2^.

 The current research note has sufficient details of the employed methods and analysis. The details provided are enough to allow for replication of the same study by others. However, there is not enough information provided regarding data presented on Tables 1 and 2. Some abbreviations used are not explained at all. The text does not at any point call to the tables. This makes it extremely difficult for non-specialists to understand the data presented there.

 The above caveat notwithstanding, most data seem to be available except for the kdr 1014F data that has not been provided ostensibly due to being part of another study. I am not sure this is good enough reason and whether or not the data could be made available at a later stage should one require them for the purpose.

 Looking at the magnitude of the question that the study is trying to address, the study falls into a situation where the results presented here simply provides preliminary information that may not fully address whatever conclusions are being proposed.

- The epidemiological significance of the East African mutant gene, 1014S kdr is not addressed here. Based on previous observation that the West African mutant 1014F kdr confers greater phenotypic resistance, it remains to be seen as to what the East-kdr spread into West Africa adds to the current resistance status. The situation becomes more complex given that all such mutations were found to be paired resulting into double mutations in all nine occurrences observed.
- The statement that *“If pyrethroids were to lose most of their efficacy, more than 55% of the benefits of vector control would be lost, leading to approximately 120,000 deaths that could not be averted”* sounds unnecessarily too alarmist! Since pyrethroids are mainly used for net treatment, the role of the net physical barrier cannot be underestimated. Moreover, the portfolio of new insecticides that are coming up should be able to avert a good proportion of the malaria burden despite the observed shortcomings by the authors.  

 Contrary to what would have been expected from such a brief research note, the authors do not attempt to make a case for further surveillance to monitor the East-kdr prevalence in the area. This is an interesting area that is developing and has not been fully explored. Previous studies and the current study have limitations in terms of time and scale, thus calling for a larger more elaborate study to establish the status of not only the spread of the East-kdr, but also its epidemiological significance in terms of its impact on phenotypic pyrethroid resistance in the country and whole of West Africa region.

I have read this submission. I believe that I have an appropriate level of expertise to confirm that it is of an acceptable scientific standard.

## References

[1] ChouaïbouMKouadioFBTiaEDjogbenouL: First report of the East African kdr mutation in an Anopheles gambiae mosquito in Côte d'Ivoire. *Wellcome Open Res*.2017;2: 10.12688/wellcomeopenres.10662.1 8 10.12688/wellcomeopenres.10662.1 28317032PMC5351847

[2] FodjoBKKoudouBGTiaESaricJN'driPBZohMGGbaCSKropfAKesseNBChouaïbouMS: Insecticides Resistance Status of An. gambiae in Areas of Varying Agrochemical Use in Côte D'Ivoire. *Biomed Res Int*.2018;2018: 10.1155/2018/2874160 2874160 10.1155/2018/2874160 30402467PMC6196986
